# Nanosonosensitizers for Highly Efficient Sonodynamic Cancer Theranostics: Erratum

**DOI:** 10.7150/thno.67168

**Published:** 2021-10-02

**Authors:** Ju Huang, Fengqiu Liu, Xiaoxia Han, Liang Zhang, Zhongqian Hu, Qinqin Jiang, Zhigang Wang, Haitao Ran, Dong Wang, Pan Li

**Affiliations:** 1 Institute of Ultrasound Imaging, The Second Affiliated Hospital of Chongqing Medical University, Chongqing 400010, P. R. China;; 2 Department of Ultrasound, Zhongda Hospital, Southeast University, Nanjing 210009, P. R. China;; 3 Department of Ultrasound, The First Affiliated Hospital of Chongqing Medical University Chongqing 400010, P. R. China.

The authors apologize that the original version of this paper 1 unfortunately contained some incorrect images. Due to negligence in drafting and proofreading, a number of similar pictures were mistakenly selected for Figure 7E, Figure 8A and Figure 8D during the figures assembly. We sincerely apologize for any inconvenience that these errors may have caused. The correct images are shown below.

## Figures and Tables

**Figure 1:**
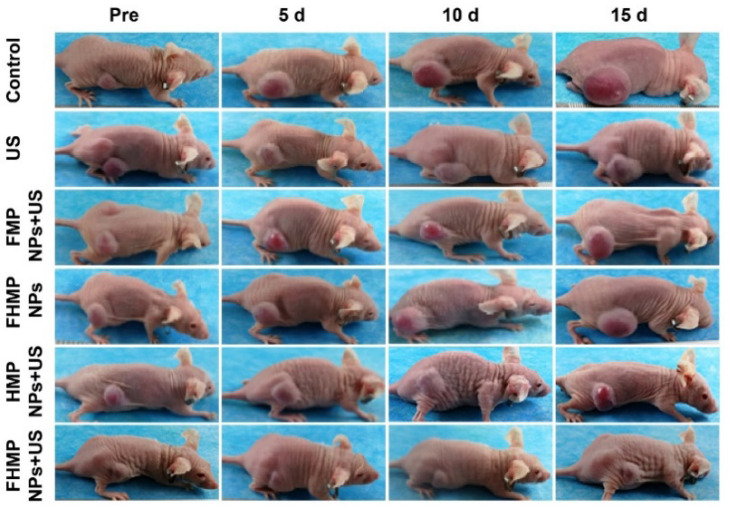
Corrected image for original Figure 7E.

**Figure 2:**
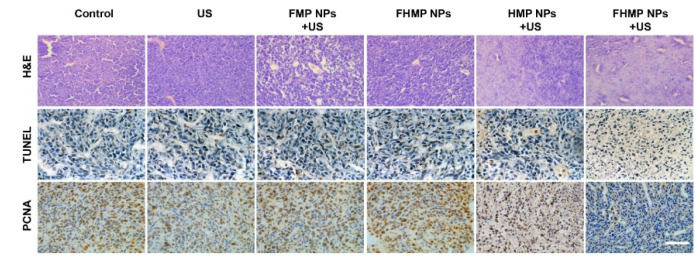
Corrected image for original Figure 8A.

**Figure 3:**
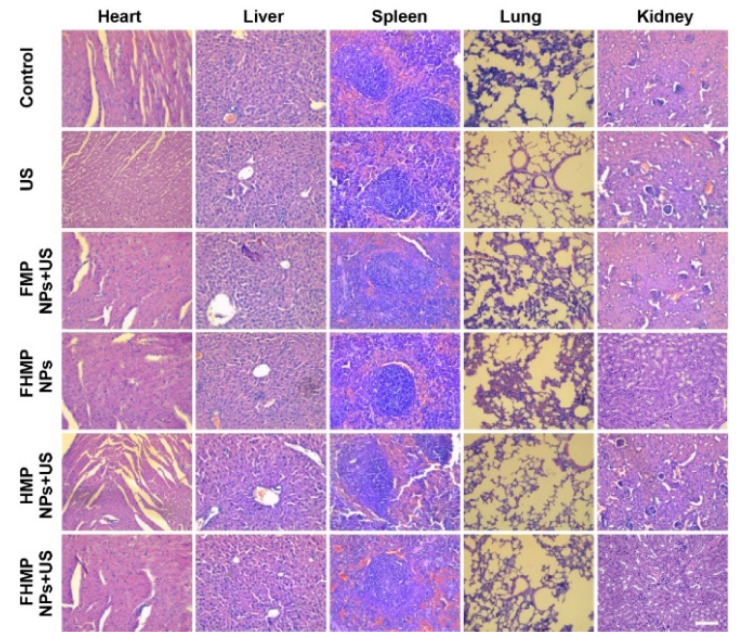
Corrected image for original Figure 8D.
